# Draft Genome Sequences of Four Strains of Recently Established Novel Veillonella Species Isolated from Human Oral Cavities

**DOI:** 10.1128/genomeA.00259-18

**Published:** 2018-04-12

**Authors:** Izumi Mashima, Yu-Chieh Liao, Amarpreet Sabharwal, Elaine M. Haase, Futoshi Nakazawa, Frank A. Scannapieco

**Affiliations:** a Japan Society for the Promotion of Science, Chiyoda-ku, Tokyo, Japan; b Department of Oral Biology, School of Dental Medicine, University at Buffalo, The State University of New York, Buffalo, New York, USA; c Department of Microbiology, School of Dentistry, Health Sciences University of Hokkaido, Ishikari-Tobetsu, Hokkaido, Japan; d Division of Biostatistics and Bioinformatics, Institute of Population Health Sciences, National Health Research Institutes, Zhunan, Miaoli County, Taiwan

## Abstract

Veillonella species are known to contribute to the formation of early oral biofilms and tend to be prevalent in people with poor oral hygiene status. Here, we report the draft genome sequences of 4 oral Veillonella strains that were established recently as novel species.

## GENOME ANNOUNCEMENT

The genus Veillonella consists of small strictly anaerobic Gram-negative cocci that lack flagella, spores, and a capsule (1). Veillonella species are isolated frequently from human oral cavities (2–5) and may serve as a biological indicator of poor oral hygiene status (6). Furthermore, Veillonella species, with their unique physiology, play a central role in early oral biofilm formation, along with oral Streptococcus species (7–10). However, the mechanistic details of their pathogenicity or functions in oral biofilms have not been clarified.

Veillonella denticariosi, Veillonella rogosae, Veillonella tobetsuensis, and Veillonella infantium were isolated from carious dentin, supragingival plaque from children, tongue biofilm from adults, and tongue biofilm from a child, respectively, and were established recently as novel species (11–14). To facilitate the study of oral Veillonella spp., the draft genome sequences of V. denticariosi JCM 15641^T^, V. rogosae JCM 15642^T^, V. tobetsuensis Y6, and V. infantium JCM 31738^T^ (= TSD-88^T^) were determined in this study.

The genomic DNA of all 4 strains was extracted from 5-day cultures using phenol-chloroform extraction and ethanol precipitation (15) and further purified using the QIAamp DNA minikit (Qiagen) for high-throughput sequencing, as described previously (4, 5). DNA libraries were prepared using the Nextera DNA library preparation kit (Illumina). DNA sequencing was performed at the New York State Center of Excellence in Bioinformatics and Life Sciences (UB Genomics and Bioinformatics Core, Buffalo, NY) using the Illumina NextSeq 500 analyzer with sequencing runs for paired-end sequences, which achieved 150-bp read lengths and over 100-fold genome coverage. The paired-end sequencing reads were checked for quality, *de novo* assembled, and annotated using MyPro, a software pipeline for prokaryotic genomes (16). At the same time, all final assemblies were annotated using the NCBI Prokaryotic Genome Annotation Pipeline version 4.4 (https://www.ncbi.nlm.nih.gov/genome/annotation_prok/) and then submitted to NCBI.

The genome sequences of the strains were assembled into 8 to 15 contigs and were nearly 2 Mb in size. These draft genomes contained, on average, a G+C content of 39.7%, 1,865.5 coding sequences (CDSs), 47.5 tRNAs, and 7 rRNAs (Table 1).

**TABLE 1:** Characteristics of 4 oral Veillonella draft genome sequences

Strain	G+C content (%)	Size (Mb)	No. of contigs	No. of CDSs	No. of tRNAs	No. of rRNAs	Accession no.	
							DDBJ	GenBank/DDBJ/EMBL
*V. denticariosi* JCM 15641^T^	42.9	1.98	8	1,801	49	7	DRA006201	PPDB00000000
*V. rogosae* JCM 15642^T^	38.9	2.19	15	1,982	48	9	DRA006205	PPCX00000000
*V. tobetsuensis* Y6	38.5	2.04	14	1,849	45	7	DRA006207	PPDF00000000
*V. infantium* JCM 31738^T^ (= TSD-88^T^)	38.6	2.02	15	1,830	48	5	DRA006199^*a*^	PPDD00000000

a Originally published by Mashima et al. (14).

To our knowledge, the annotated genome sequences of V. denticariosi, V. rogosae, and V. infantium presented here are the first ones available. The draft genome sequence of the V. tobetsuensis type strain was reported in our previous study (17). These data should be helpful in future studies of the biology and pathogenicity of oral Veillonella spp.

### Accession number(s).

The raw sequence data were deposited to the DDBJ Sequence Read Archive (SRA), and draft genome sequences were deposited to GenBank/DDBJ/EMBL under the accession numbers listed in Table 1. The versions listed in this paper are the first versions.
